# A Comprehensive Review on Edge Caching from the Perspective of Total Process: Placement, Policy and Delivery

**DOI:** 10.3390/s21155033

**Published:** 2021-07-24

**Authors:** Honghai Wu, Yizheng Fan, Yingda Wang, Huahong Ma, Ling Xing

**Affiliations:** 1 School of Information Engineering, Henan University of Science and Technology, Luoyang 471000, China; 190319050234@stu.haust.edu.cn (Y.F.); mhh@haust.edu.cn (H.M.); xingling_my@haust.edu.cn (L.X.); 2 School of Computer Science and Technology, Shandong University, Qingdao 266200, China; 201920130246@mail.sdu.edu.cn

**Keywords:** MEC, edge caching, caching placement, delivery process, D2D

## Abstract

With the explosive growth of smart devices and mobile applications, mobile core networks face the challenge of exponential growth in traffic and computing demand. Edge caching is one of the most promising solutions to the problem. The main purpose of edge caching is to place popular content that users need at the edge of the network, borrow free space to reduce user waiting time, and lighten the network load by reducing the amount of duplicate data. Due to the promising advantages of edge caching, there have been many efforts motivated by this topic. In this paper, we have done an extensive survey on the existing work from our own perspectives. Distinguished from the existing review articles, our work not only investigates the latest articles in this area, but more importantly, covers all the researches of the total process of edge caching from caching placement optimization, policy design, to the content delivery process. In particular, we discuss the benefits of caching placement optimization from the perspective of different stakeholders, detail the delivery process, and conduct in-depth discussions from the five phases, i.e., requested content analysis, user model analysis, content retrieval, delivery, and update. Finally, we put forward several challenges and potential future directions, and hope to bring some ideas for the follow-up researches in this area.

## 1. Introduction

With the exponential growth of mobile data traffic and the increasing scarcity of energy and bandwidth resources [1], to meet the denser service requests in mobile networks, more fine-grained content delivery network (CDN) technology and more efficient caching strategies are required. Various networking services are springing up, which also increases the demand for high-quality content access, inevitably placing a heavy burden on the core network and backhaul. Moreover, the main technology in the era of centralized big data processing with cloud computing as the core can no longer efficiently process data generated by edge devices. Currently, ultra-wideband communication [2], large-scale multi-input multi-output (MIMO) communication [3], millimeter-wave communication [4], and heterogeneous network [5] have been proposed, which are several important technologies applied to wireless communication systems. All of these technologies require an expensive backhaul link between the base station (BS) and the core network (or other BSs) to reduce backhaul traffic [5,6,7]. Therefore, managing an overloaded network is also an important current issue, and a new network framework is urgently needed to solve it.

**Figure 1:**
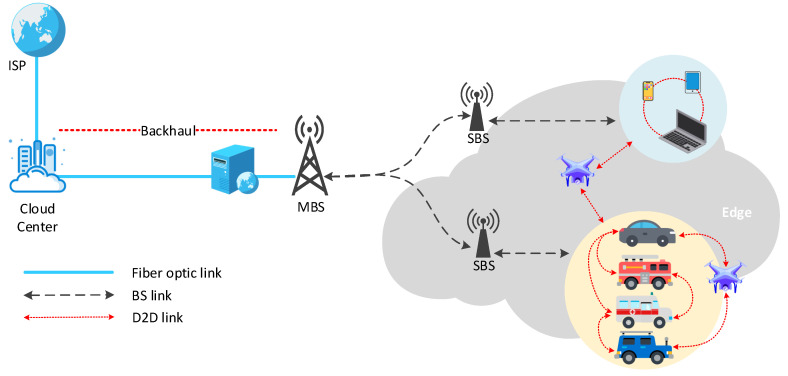
Architecture of MEC.

Mobile cloud computing (MCC) improves the performance of mobile devices (MDs) [8] with the assistance of cloud computing and delivers applications directly to mobile devices with cloud computing and storage functions [9]. However, bandwidth constraints and network congestion limit the exchange of big data between multiple users and the central cloud at the same time [10]. Thus, mobile edge computing (MEC) based on distributed design is proposed to make computing tasks and content distribution closer to terminal devices and users [11]. As shown in Figure 1, the macro BS (MBS) and the cloud center are connected through a fiber optic link, the MBS and the small BS (SBS) are connected through a BS link, and the edge devices are connected through a device-to-device (D2D) link. With this advantage, MEC can greatly reduce service delay [12] and offload traffic from the backhaul. At the same time, MEC has also expanded the coverage and capacity of the mobile network, which is conducive to the load balancing of the backhaul link, and dynamically invokes computing and storage resources according to user needs, thereby reducing the energy consumption of mobile terminals and extending the life of the network [13,14]. Caching technology, which relieves most of the pressure of wireless network transmission by reusing Internet content, has also become an effective tool to reduce the peak data rate. It is a more promising solution to alleviate the above problems by using caching technology in MEC and enabling terminal devices to have the caching capability. Edge caching has advantages of CDN [15], and then using an MEC server as an edge cache node can dynamically optimize content delivery services based on the network status and wireless channel status [12], providing mobile users with high availability and more recent content and services [16].

Since it is expected to solve a series of problems caused by limited spectrum and bandwidth resources, edge caching technology has also become one of the hot topics in the research of MEC, and extensive researches have been done. Some researchers have conducted investigations in their work. The authors of [12] mainly investigated the four key research issues of mobile edge computing architecture, computing migration, edge caching, and service orchestration. However, their work in edge caching only involves caching performance, measuring content popularity, and some caching strategies. They have not summarized caching strategies detailedly and lack a comprehensive investigation of the caching process. The authors of [17] compared the local cache between the wired and wireless edges from the physical layer and the media access control (MAC) layer, but the authors did not pay attention to the choice of cached contents and the update of cached contents. The work of Goian et al. [18] focused on the caching technology of popular content, providing a comparison between traditional and popularity-based caching, and thus proposed that popularity prediction in edge caching is crucial for service providers and users. However, their investigation is limited to some active caching work, focusing on the prediction of caching content. Yao et al. [19] conducted a detailed investigation on mobile edge caching and compared different caching policies according to the impact of different caching locations and different caching performance indicators on caching strategies. However, they did not discuss the impact of users’ social attributes on the edge caching separately, nor did they pay attention to the different stakeholders in the edge caching. This motivates us to survey the current methodologies and present the corresponding challenges and potential research directions. We have investigated lots of related articles published in the past five years, most of which focused on caching placement optimization, policies and delivery processes, and selecting articles with high relevance and significant contributions in this field to investigate the edge caching comprehensively.

In theory, improving the performance of the edge caching system requires consideration of where the cache is, how to cache, and how to deliver cached content to users. Our work investigates existing research from caching placement, policies, and the delivery process, covering the total caching process. This article first discusses the benefits of caching placement optimization from the perspective of different stakeholders, and then compares the pros and cons of different caching policies. In particular, our work also focuses on the entire delivery process and conducts in-depth discussions from the five phases, including requested content analysis, user model analysis, content retrieval, delivery, and update.

The rest of the survey is organized as follows. This work is mainly a review of several important parts of the edge caching: we summarized three different caches storage locations (Section 2), introduced several different caching schemes (Section 3), and sorted out related work in the four main stages of caching delivery (Section 4). Section 5 discusses the main challenges facing edge caching and possible future research directions. The investigation ends in Section 6.

## 2. Placement Optimization

**Figure 2:**
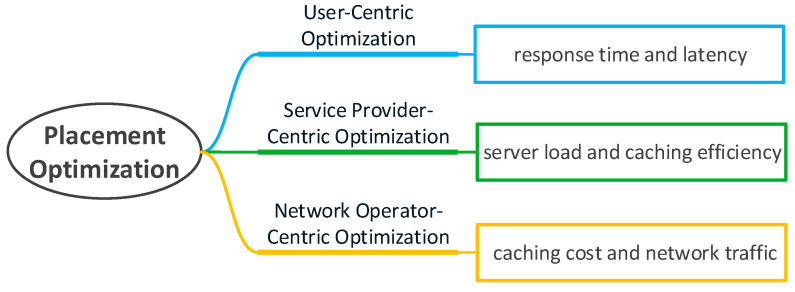
Placement optimization.

**Figure 3:**
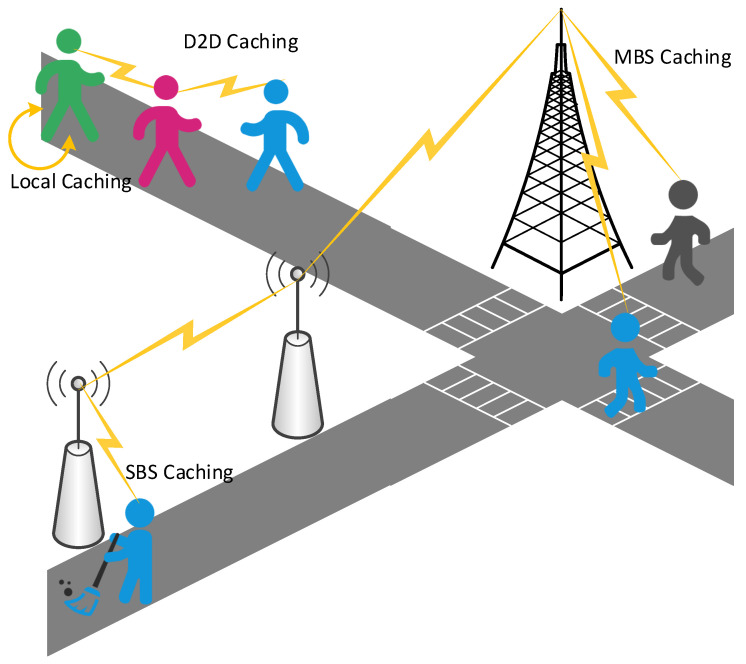
Different caching locations.

In order to reasonably allocate caching resources at the edge of the network and maximize the use of edge nodes, many scholars have conducted research on caching placement. However, choosing an appropriate location as the caching node in the edge caching is affected by multiple factors, and there is no general strategy. Therefore, how to optimize caching placement to effectively solve these problems has become a focus of edge caching. In particular, in the recent work we investigated, we have noticed that the indicators that affect the optimization of caching placement usually include latency, caching efficiency, and caching cost. Related work requires quantitative analysis of the information that affects these indicators. For example, the hedge algorithm is used to quantify the time for the caching node to access the storage sector. Integer coding is used to quantize the requested file information. An effective capacity-based utility is introduced to quantify the end-to-end user-perceived delay and date rates. In addition, a regret bound is used to quantify the expected cache benefits and so on. Thus, we will classify these works from the perspective of different stakeholders. As shown in Figure 2, the users’ concern is whether the sent request can be responded to within the expected time, service providers are more concerned about the problems of server load, and network operators need to consider how to reduce cache costs and network traffic. We will discuss from the three perspectives of users, service providers, and network service providers. Edge caching at different locations is shown in Figure 3, which are cached at different base stations (BSs) and MDs.

### 2.1. User-Centric Optimization

From the benefit of users, in the work of optimizing the placement problem, more attention is paid to reducing the response time and delay of user requests and improving the fairness between users. Users on the move usually have short connection durations and frequent handoffs, making video streaming suffer more delays from handoff and connection. In order for mobile users to enjoy higher quality services of video streaming, Qiao et al. [20] proposed a cache-based millimeter wave framework, which uses the Markov decision process to dynamically allocate BSs cache space to mobile users, and then reduce the computational complexity of dynamic programming to reduce latency. When some works take into account user selfishness, the differences between various content and the impact of user mobility are ignored easily. Zhang et al. [21] proposed a heuristic caching placement algorithm based on multi-winner repeated auctions, which reduces caching redundancy and reduces the average content access latency. In order to reduce the average download delay of users, Liu et al. [22] considered flexible physical layer transmission schemes and content preferences of different users and proposed a transmission-aware caching placement strategy. Experiments show that this strategy can achieve an average delay close to the centralized mode in the distributed mode. However, they did not consider the user spatial location and activity level. Chen et al. [23] considered the size of the available caching space and the distance between the caching node and the destination. Four placement strategies are used: Uniform, Weighted, Mid, and Nearest. However, they rely on the shared local information of caching nodes without considering the security of users’ data. Considering that the user mobile information may help reducing network latency, Sun et al. [24] proposed a mobility-aware caching placement problem. They transformed the problem into a multi-phase decision-making problem, solved it through dynamic programming and used the recursive relationship of adjacent phases, and then got the optimal caching strategy. This strategy can significantly reduce the average file delivery delay when the user moves. Tran et al. [25] minimized the average residence time of all users in the caching system under the constraints of the known storage size of BSs and the delay tolerance of requesting users. Jiang et al. [26] proposed a caching placement algorithm based on greedy methods to find peer nodes in the caching process, and reduce the transmission delay through a cooperative strategy. They also established a dynamic optimization model based on peer-to-peer selection algorithms to protect the privacy of matching data.

However, they all ignore the fairness between users, which is also very significant for improving user experience during the caching process. Facing more complex content delivery and placement caused by flexible users and BSs association, Jing et al. [27] proposed an iterative association-aware content placement algorithm. The algorithm can better deal with users of different activity levels and improve the fairness of the caching. In order to improve fairness and minimize the average download time, Li et al. [28] considered the limited storage space and connection capacity of the caching node and proposed an algorithm based on the alternating direction method of the multiplier. This algorithm can effectively search for the caching location and improve the effective utilization of the storage space of caching nodes and the efficiency of caching transmission. However, the connection capacity of caching nodes and the available storage space may change.

### 2.2. Service Provider-Centric Optimization

For the benefit of service providers, in the work of optimizing placement problems, more attention is paid to reducing server load (computational complexity, communication distance and hop count, etc.) and improving caching efficiency (caching hit rate, caching redundancy, etc.). In the early days, researchers put forward the concept of “less for more”, which is to select a suitable caching node to cache data for a specific request path, and proposed a caching strategy based on the concept of betweenness centrality to get the maximum caching hit rate [29]. After that, Wang et al. [30] studied the problem of cache allocation in routers with storage functions, and they found that there are many factors that affect cache allocation, and existing caching strategies cannot be completely balanced. In order to reduce the average hop count requested by users and caching operations on the router, Hu et al. [31] considered content popularity, hop reduction gain, penalty of caching space replacement, and the combination of caching replacement and location, and proposed a low complexity of the caching placement scheme. By taking into consideration application specific parameter, Parvez et al. [32] proposed a caching node placement scheme based on a genetic algorithm, in which the caching system determines the active caching node and the auxiliary caching node, and also considered the transmission range of each active caching node. Parrinello et al. [33] studied how the dedicated caching placement can achieve the best state. Based on their work, Asadi et al. [34] proposed a placement strategy based on incremental caching to minimize the load of the shared link in the worst case. However, they all ignored the changes in the network topology. In order to optimize the number of user hops and the balance of node load, Shan et al. [35] proposed a caching placement scheme based on a particle swarm optimization algorithm, which can select the best caching location in various network topologies. Due to the characteristics of Information-Centric Networking, the data copy rate is too high and the cache space cannot be fully utilized. Wang et al. [36] designed a caching location selection algorithm based on the Pareto model to solve the problem of excessive caching redundancy. But the research in this article is limited to solving the problem of caching space.

Taking the communication distance and the number of communication hops as reference indexes for selecting the caching location can also effectively reduce the load of BSs and improve the caching efficiency. Okada et al. [37] calculate the communication volume between users and caching nodes from the access probability and the number of communication hops, and select the caching location based on the communication volume. On the Internet of Vehicles (IoV), Bitaghsir et al. [38] proposed a caching placement algorithm based on Multi-Armed Bandit Learning, which selects the content to be cached in the roadside unit (RSU) according to the content popularity, and then uses the user social characteristics to select the optimal caching path. This algorithm effectively reduces the load of each caching node. Considering that D2D communication is between adjacent users, the optimal caching location will change as the user moves. Song et al. [39] transformed the minimization of the average data load of a BS problem into a problem of maximizing sub-modular functions with matroid constraints and then used a greedy algorithm to obtain a solution close to the optimal caching performance. They significantly reduce the average load of BS in the D2D caching. Service providers also need to consider the performance of overall placement performance. In [40], the robust information entropy is used to evaluate the overall performance of sensor placement and avoid some possible identifiability problems. In subsequent work, service providers can also use information entropy to evaluate the optimal placement decision.

### 2.3. Network Operator-Centric Optimization

For the benefit of network operators, in the work of optimizing placement problems, the purpose is more inclined to reduce cache costs (including opening up cache space) and network traffic. Maddah-Ali et al. [41] proposed that the joint caching placement and delivery phase to optimize the caching system can reduce the total data transmission, thereby significantly improving caching gain and reducing caching cost. Because appropriate placement mechanisms are needed to improve sever latency while still minimizing cost, Ghalehtaki et al. [42] proposed a bee colony-based algorithm for micro-cache placement through the virtual network function to realize a micro-cache in closer caching nodes. Zou et al. [43] decoupled the problem of maximizing the interests of network operators from the available storage space of the BS into a set of knapsack problems, and then proposed an iterative dynamic programming algorithm based on the Stackelberg game. Their experimental results show that network operators can obtain higher profits by using collaboration between BSs. To maintain caching costs under budgets in the long run, Gao et al. [44] formulated the caching placement problem as a combinatorial Multi-armed Bandit (MAB) problem with long-term time-average constraints when the content popularity is unknown and proposed an auxiliary caching placement scheme that combines online learning and online control. This solution can control the caching cost for a long time, thereby maximizing the quality of service (QoS) gain.

### 2.4. Summary and Discussion

However, from any stakeholder’s perspective, the ultimate goal of caching placement optimization is to improve the overall performance of the caching system, so that all three parties benefit from it. We have sorted out caching placement from three different perspectives, and most of the work is focused only on the interests of one party to select the most appropriate caching node. They have solved some problems separately, but there are still many challenges to be overcome urgently. In order to reduce the probability of outage, inter-cell signal interference of BSs near users, cache size, deployment density of small BSs (SBSs), and spectrum allocation all need to be considered. In dynamic scenarios, it is necessary to consider the mutual coordination between caching nodes to avoid more path loss affecting caching performance. Moreover, changes in the network topology seriously affect the optimization effect of caching placement, and how to deal with this challenge is still a difficult problem. Finally, how to better weigh the interests of users, service providers and network service providers needs to be explored all the time. In addition, the performance of the caching strategy also depends on the location and number of caching nodes. Similar to the work of [45] on optimizing sensor placement, using entropy to quantify the effect of caching placement may result in a more compromised decision.

## 3. Caching Policies

**Figure 4:**
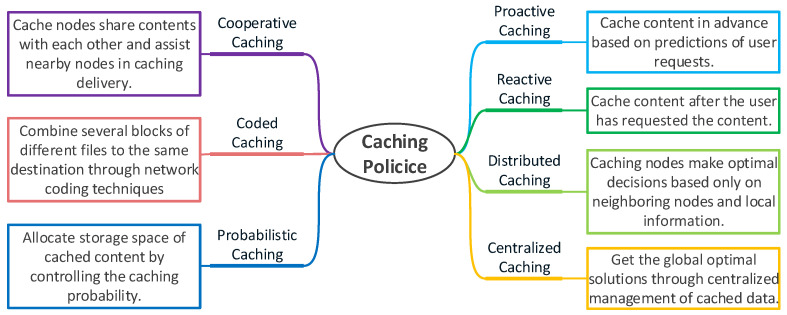
Caching policies.

When caching content at edge nodes, in order to better adapt to different scenarios and performance requirements, different caching policies are proposed. We divide the existing caching policies into five classes based on their different characteristics, as shown in Figure 4, and then compare their pros and cons.

### 3.1. Proactive and Reactive Caching

Reactive caching is to determine whether to cache the content after the user has requested the content [19]. Proactive caching is based on the current network traffic dynamics, actively storing popular content in selected caching nodes during off-peak periods, thereby alleviating network traffic pressure [46]. Bastug et al. [46] proposed a proactive popularity caching algorithm, which caches the most popular content in a limited storage unit according to the popularity ranking of the requested content. The algorithm improves system performance by satisfying the number of requests and reducing traffic, and proves that proactive caching is better than reactive caching. The author also uses the user social network and D2D communication to design a proactive caching policy based on the content popularity following the Zipf distribution [47].

Proactive caching can be very costly to meet the needs of users during the peak period. In order to maintain long-term load balance and make full use of storage resources, Tadrous et al. [48] used the predicted user needs and the computing and storage resources provided by MDs to smooth wireless network traffic, thereby improving the quality of proactively cached contents and reducing system costs. However, the accuracy of predicting content popularity and user behavior is the biggest challenge facing proactive caching, and inaccurate prediction information will seriously affect the performance of the caching system [49]. In large-scale caching of heterogeneous networks, proactive caching randomly can provide the diversity of cached files, thereby improving network performance. At the same time, the use of BS joint transmission can increase the probability of the user successfully receiving the requested content [50]. Wen et al. [51] proposed a proactive caching policy using random discontinuous transmission (DTX) for retransmission. Experimental results show that this scheme is superior to the existing baseline scheme and can make more reasonable use of storage resources and transmission opportunities when system parameters change. Hu et al. [52] studied a random caching strategy based on the cooperation radius and designed the best caching strategy by estimating the file size and Zipf index. The author also continues to study the random caching strategy based on cooperation radius in heterogeneous networks with random DTX, and the best caching strategy obtained can stably achieve better average successful transmission probability (STP) performance, and is not affected by content popularity distribution [53]. The traffic increase is caused by duplicate routes of cache transfer from a storage location to caching locations and by caching placements on more caching nodes to improve caching hit rate according to MD transition, which will also lead to an increase in system latency and energy consumption. Tanaka et al. [54] used routing deduplication technology in the proactive cache system for caching transmission and selectively performed data transmission from the layout and distribution of MDs. At the same time, predictions are made through cellular radio information to reduce traffic between edge servers.

Since the accuracy of predicting user behavior and content popularity has a great impact on the performance of the caching system, a lot of work of proactive caching requires accurate prediction information. How to achieve sufficiently accurate prediction accuracy is still a difficult problem.

### 3.2. Distributed and Centralized Caching

Centralized caching is the centralized management of cached data. The central controller monitors the global network status and analyzes the channel status information and user information to make caching decisions after receiving the request. Cui et al. [55] proposed a centralized control caching strategy based on content popularity and centrality. The central controller obtains information through the control node, controls different nodes to collaborate effectively, and reduces the number of hops from the control node to the caching node. This minimizes the path from the user to the server, thereby increasing the caching hit ratio and reducing the average transmission delay [56]. With the surge in user business requests, the central controller is faced with a large number of service needs to be processed, which also puts a great burden on the link between the server and the edge node and affects network efficiency. Distributed caching is the key to breaking this bottleneck, which allows caching nodes to make optimal caching decisions based only on neighboring nodes and local information. Distributed storage of service data in cache-enabled BSs can reduce traffic pressure on future mobile networks. With limited storage space, BSs must update the cached content according to changes in content popularity to obtain better caching efficiency [57]. Wang et al. [58] considered the trade-off between the diversity and redundancy of BSs’ cache and proposed a distributed caching policy. They use an adaptive particle swarm algorithm to obtain the best redundancy rate under a given system configuration and minimize the total cost of network transmission. Through the interdependence between the caching strategy and the physical layer coordination, Ao et al. [59] proposed a system framework that combines distributed caching of small cells and coordinated transmission of neighboring BSs to maximize the caching hit ratio. Their proposed zero forced beamforming is used for multiple users to achieve multiplexing gain, allowing joint cross-layer optimization in the system to achieve a faster content delivery speed.

The belief propagation method has been used to solve the problem of resource allocation in mobile networks. Therefore, many researchers use the belief propagation algorithm to optimize distributed caching. In order to meet the needs of different users in the mobile network for popular content on different networks and solve the problem of file placement in the distributed caching, Li et al. [60] designed a belief propagation algorithm for optimizing distributed caching and proposed a distributed belief propagation algorithm with the help of network factor graphs. Simulation results show that the algorithm can achieve the best delay performance of exhaustive search within a small margin, and the improved heuristic belief propagation algorithm can provide good delay performance under low communication complexity. The work of Liu et al. [61] provides multiple caching BSs for each user, and the distributed belief propagation algorithm proposed therein can minimize the average download delay of the system. In this algorithm, each BS performs calculations by analyzing the collected local information and reduces the iterative exchange of information between neighboring BSs until the calculation results converge. Simulation results show that the algorithm can significantly improve the performance of content delivery in distributed caching.

In the fog wireless access network, Lu et al. [62] considered the popularity of unknown temporal and spatial content and user preferences to study the distributed edge caching problem. They established a user request model through a hidden Markov process, proposed a Q-learning method based on reinforcement learning and a distributed search for optimal caching strategy, and used fog access points to learn and track dynamic processes, avoiding additional communication overhead. In the ultra-dense fog wireless access network, Hu et al. [63] considered time-varying user requests and ultra-densely deployed fog access points. They proposed a dynamic distributed edge caching policy, which uses network characteristics to approximate the random differential game to a mean-field game, and makes dynamic caching decisions based on local information. The simulation results show that this scheme can reduce request service delay and forward traffic load.

When BSs do not belong to the same service provider, centralized solutions are difficult to achieve. Distributed solutions can respond to local changes faster and have less impact on other nodes’ caching decisions. However, due to the lack of analysis of the global network status, distributed solutions often fail to obtain the best results, so the distributed caching strategy still needs to continue further research to ensure the overall performance of the system.

### 3.3. Cooperative Caching

To alleviate the storage capacity limitation of edge caching nodes, efficiently use the idle period of the storage capacity in some caching nodes, and solve the problem that the limited capacity of caching nodes affects caching efficiency through a collaborative caching strategy, Yang et al. [64] considered the cooperative caching of relay nodes and users in their work, and proposed a wireless cooperative caching strategy to minimize network transmission energy consumption. In the process of caching node collaboration, the delay of retrieving content also needs to be considered and excessive signaling of overhead information when acquiring the cache status of nearby nodes. In order to minimize the overhead of collaborative caching, Jiang et al. [65] used Femto BSs to collaborate with users for content caching and delivery. The cooperative caching problem is approximated as an integer linear programming problem, the gradient algorithm is used to obtain the optimal solution, and the Hungarian algorithm is used to solve the problem of unbalanced distribution in content delivery. This strategy can significantly reduce redundant data transmission while improving content distribution performance. Li et al. [66] proposed a collaborative caching placement framework, which reduces the content transmission time of mobile users through coordinated multi-point joint transmission and also converts the optimization problem into a local altruistic game. The simulation results prove that the algorithm can reach the global optimum with a high probability and converge faster, significantly reducing the content transmission time of mobile users.

Research on collaborative caching strategies also needs to consider the collaboration costs of different content providers and users. Content providers need to pay mobile network operators to cache content in BSs, and user’s devices also need to consume a certain amount of storage capacity and energy consumption when storing and transmitting contents. Considering collaborative caching from an economic point of view, Gharaibeh et al. [67] studied minimizing the total cost paid by content providers in a multi-unit collaborative system and proposed an online caching algorithm that made caching decisions from content cached in nearby BSs or retrieved content from the Internet, eliminating the need to estimate content popularity. The simulation results show that the scheme saves more caching payment costs, and at the same time, it is still better than the offline collaboration scheme in the case of estimating content popularity. However, their system does not consider the storage capacity limitation of BSs and the content popularity and assumes that the cost of obtaining content from BSs is lower than obtaining content from the Internet. Ostovari et al. [68] studied the problem of minimizing the cost of the content provider in collaborative caching based on content popularity. They took advantage of the sub-module attribute of the problem and proposed an offline algorithm, assuming that content popularity is a priori, the objective function is to minimize the sub-module function, and the greedy algorithm is used to approximate the solution. At the same time, paper [68] quoted online algorithms similar to paper [67], and used random linear network coding to find the best solution to measure the performance of online algorithms.

**Figure 5:**
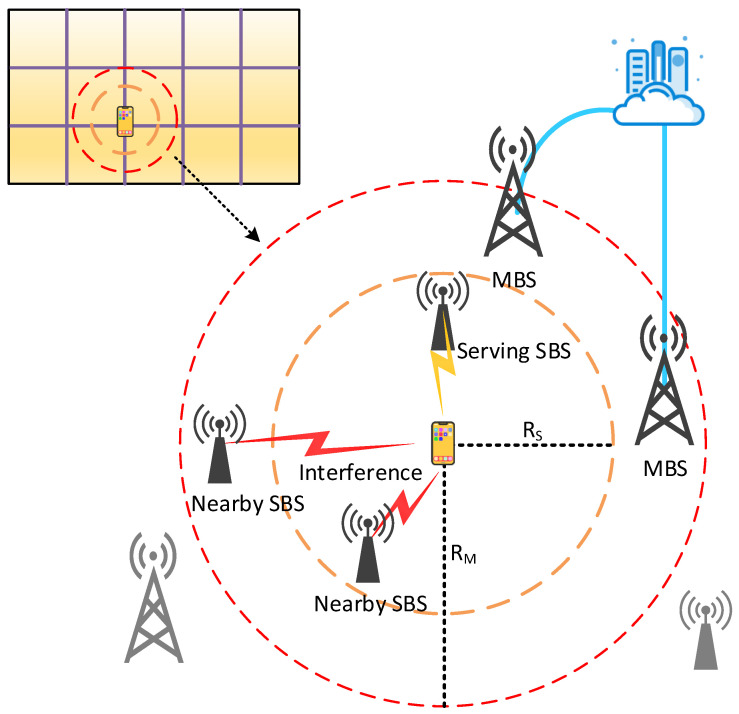
Cache-enabled user-centric network.

To make more effective use of the limited caching capacity, Chen et al. [69] divided SBSs into disjoint clusters as caching entities and proposed a combined caching policy in which part of the caching space in the cluster is used to cache currently popular content, and the remaining space was used to collaboratively cache different parts of less popular content. Zhang et al. [70] studied the optimal delay of cooperative edge caching in large-scale user-centric mobile networks. They used the state information of random information to optimize cache placement and cluster size and proposed a greedy cache placement algorithm based on bandwidth allocation to balance content diversity and spectrum efficiency. As shown in Figure 5, user-centric network organizes a dynamic BS group for each MD, and the range of the BS group is the radius RS to RM. Chen et al. [71] used the random set method to derive the relationship between the average outage probability and the BS-to-MD density ratio, cache size and BS group radius, and found the optimal caching distribution. They optimized the lower bound of the average outage probability under the constraint of cache size and obtained higher performance gains from BSs within the BS group.

The cooperative caching strategy solves the problem of the capacity limitation of caching nodes. However, the cost of collaboration between content providers and users needs to be considered, and a reasonable incentive mechanism should be designed. At the same time, how to compress the time for retrieving a cache in the process of caching node collaboration and how to reduce the additional signaling overhead required to obtain the status of nearby nodes is still a problem to be solved in the current research work.

### 3.4. Coded Caching

The model of the caching network consists of a single server connected to multiple cache-enabled servers through a broadcast channel. The server obtains global caching gains by encoding multicast transmission and can serve multiple users at the same time. The global caching gain is the only caching gain that varies with system parameters. Therefore, it is the basic amount of the cache network, and the coded multicast transmission is an important technology in the caching network [72]. Encoding plays a key role in the caching network. Through encoding technology, the transmitted content can be encoded at the caching node and then decoded at the target node. This can reduce the amount of data that needs to be transmitted, but it also increases the computational overhead at the caching node. However, coded multicast transmission has brought an order of magnitude improvement in bandwidth efficiency. Fadlallah et al. [73] focused on the impact of coding calculation and communication overhead on system bandwidth efficiency performance. They combined the MAC layer frame design with the Orthogonal Frequency Division Multiplexing (OFDM) physical layer to be compatible with mobile networks or other physical layer standards and allowed each receiver to decode the coded data. The author’s experimental results show that the additional coding overhead in a small-scale network will not affect the performance improvement brought by coding multicast, and this design can effectively reduce bandwidth requirements and transmission delays. The basic coding and caching policy are to divide each piece of cached content into a large number of non-overlapping sub-files and store them in servers, and they are not suitable for the case of small cached files. Tang et al. [74] proposed a coding and caching policy based on combined structures, which are obtained from linear block codes with a certain rank attribute in the generation matrix. This solution is at the cost of increasing the rate, and the sub-grouping level obtained is much lower than the basic solution. When faced with the application of various problem parameters, the number of subgroups still increases exponentially with the increase of users [75]. Most of the work has not considered that in actual networks, many caching systems are composed of multi-layer caches, arranged in a tree structure, with the origin server as the root node, and edge caching nodes as the leaf node to directly serve users [76]. Karamchandani et al. [76] proposed a hierarchical coding caching policy in a layered network with two layers of caching, using the coding multicast transmission in each layer, and providing cross-layer coding multicast opportunities between different layers. Takita et al. [77] proposed a combination of three basic caching policies based on the lower bound of the cut set boundary problem to solve the coding caching problem in a hierarchical network with multi-layer caching. However, during the peak traffic period, the current best way is to cache the content in MDs, and users can mainly transmit the requested content through D2D. In a cache-enabled MDs system, Ibrahim et al. [78] proposed a coded caching scheme that minimizes the worst-case delivery load for D2D-based content delivery to users with unequal cache sizes. They considered the situation of users with different storage capabilities to minimize the D2D transmission load under poor network conditions. However, they did not consider the feasibility and performance of the solution when the cache is not equal to the user.

The coding caching reduces the amount of data necessary to be transmitted but inevitably increases the computational overhead at the caching node. Generally, the caching system in the network is multi-layered, and the network and the number of layers may be asymmetrical. Therefore, for delivering content more effectively, coded prefetching may be an interesting research subject.

### 3.5. Probabilistic Caching

Unlike wired networks, the problem of the caching nodes layout becomes more complicated due to changes in locations and requests of users in wireless networks. In order to solve the optimization problem of caching placement, some researchers have proposed a probabilistic caching strategy, which allocates storage space of cached content by controlling the caching probability. Zhang et al. [79] proposed a probabilistic caching model to solve this problem. However, due to computational complexity, it is difficult to derive a closed-form solution. Zhang et al. [80] proposed a mathematical framework based on random geometry to represent the caching hit probability, using the Lagrangian multiplier method to solve the optimization problem of cache placement. In the multi-layer wireless heterogeneous network, consider the optimization of the probabilistic caching of all types of BSs, and solve the problem of caching placement through the probabilistic caching in different layers, which usually faces the correlation probability between different layers and the complex interference distribution in heterogeneous networks. Li et al. [81] found that the successful delivery rate in a single-layer network only depends on the cache size of BSs, while the successful delivery rate in a multi-layer network also considers the impact of BSs’ density and transmit power. By establishing a connection between the two, they proposed a probabilistic caching policy that maximizes the successful delivery rate.

The probabilistic caching strategy is more to solve the problem of caching location in the wireless caching network. The difference in cache size also reduces the caching performance and the correlation probability between different network layers, so probabilistic caching still needs to be studied continuously.

## 4. Delivery Process

**Figure 6:**
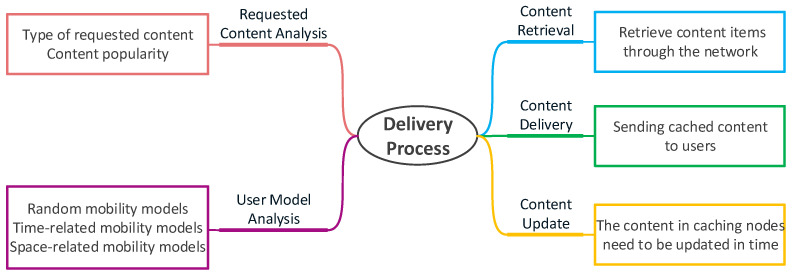
Delivery process.

**Figure 7:**
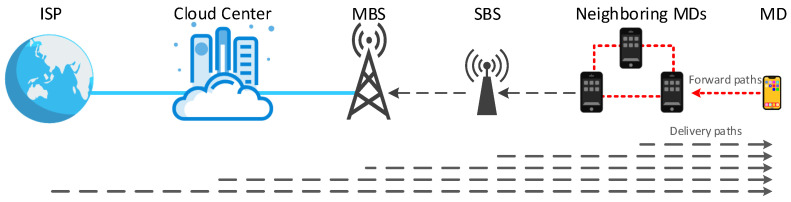
Content retrieval process.

The delivery process is the process of delivering cached content to users. It mainly includes five phases: requested content analysis, user model analysis, content retrieval, content delivery, and content update. As shown in Figure 6, the caching system first analyzes the type and popularity of the requested content and then analyzes the user model to determine an appropriate caching strategy. After that, the user needs to retrieve the required content items through the network, find the content with the help of neighboring devices or caching nodes, and determine the method of retrieving the content and the caching location, as shown in Figure 7. The next step is to choose a suitable way for content delivery. Finally, the system should consider the timeliness of cached content and design effective caching update strategies. We will discuss this from these five phases.

### 4.1. Requested Content Analysis

We first analyze the type of requested content and predict the content popularity in the future, which is more conducive to making caching decisions suitable for users. It is feasible to obtain the next possible request model from the user’s past request records, but in the actual dynamic environment, it is a big challenge to predict and simulate the user request model [82]. In this section, we discuss the cache types and popularity.

#### 4.1.1. Cache Types

In edge caching, the most common types of cached content are files and videos. From the requirements for service quality, the requested content can also be divided into two categories: (1) Real-time content that requires low latency, which needs a stringent downloading time, and (2) popular content that requires high network throughput, which need to be transmitted before a less stringent service deadline. For example, road traffic information in IoV has strict timeliness and needs to be transmitted to target users in a timely manner. Mobile users watch popular films and television shows online and have a high tolerance for transmission delay but require high network throughput to ensure high-quality and continuous content transmission.

Faced with high-speed changes in network topology in IoV, massive amounts of information need to be stored, and a large amount of onboard content is generated and updated in real-time. Processing these data services requires very high network efficiency. At the same time, the life cycle of IoV data is short, and network resources are shared among more users, so more targeted strategies are needed for resource management and caching. In order to ensure the QoS of applications and servers, Paranthaman et al. [83] used probabilistic mechanisms to improve resource management in public networks, thereby reducing user disputes over fairness. However, due to the limited storage at RSUs and soaring content size for distribution, RSUs can only selectively cache content replicas. Su et al. [84] analyzed the characteristics of the content request in the car according to the content access mode of the vehicle, the speed of the vehicle and the road traffic density, and then a cross-entropy-based edge caching scheme is proposed based on the request decisions, which can dynamically adapt to the requests of different vehicles. Considering the time-varying nature of content popularity prediction, Vigneri et al. [85] distributed different retrieval deadlines to different types of content, which can maximize the amount of offloading and reduce the impact of intensive request processing on the quality of users’ experience.

#### 4.1.2. Content Popularity

In the design of the caching strategy, content popularity is an important reference data. In the research work of content popularity, the static distribution that is artificially assumed at the beginning adopts an independent reference model. In the initial research, many researchers studied content popularity based on Zipf distribution [86]. Bastug et al. [47] utilizes the user social network and D2D communication and assumes that the content popularity follows the Zipf distribution. Zhu et al. [87] proposed that the Zipf model is not suitable in video-on-demand (VOD) and then used the drift power-law model and the extension index model to describe the video popularity in the short-term and long-term respectively. However, they did not consider the opportunities in the long tail effect of niche videos [88]. It is more convenient to establish a static model of content popularity to research, but content popularity is constantly changing over time [89]. Therefore, more research work will take into account the time-varying nature of content popularity, and the changes in the content popularity described through complex online interactions and information cascades are difficult to predict [90].

The advent of the era of big data and the rapid development of machine learning algorithms provide new support for accurately analyzing the content popularity. Wrong information will affect the probability of finding the requested content in the caching; therefore, Mehrizi et al. [91] used the Poisson distribution of the Gaussian process of content feature information to assist in enhancing prediction. Whereas, assuming that users’ demand for content follows a Poisson distribution, it can lead to deviations in the modeling process. In order to reduce this deviation, Yang et al. [92] modeled the content popularity model as a linear model, proposed an online ridge regression algorithm based on content features and location customization. Considering the unknown content popularity distribution, Song et al. [93] studied the distribution of content popularity from the perspective of MAB. Paper [94] is based on the research of paper [93], adding collaborative caching between BSs to further improve user experience. Changes in content popularity are often unstable, and the selected feature samples also need to be continuously updated [95]. Tang et al. [96] proposed a reinforcement learning-based scheme to reflect the popularity of files and user preferences, in which training samples are continuously generated through the feedback mechanism of the Markov Decision Process (MDP). Hou et al. [97] used the transfer learning-based method for long-term training with sufficient data volume, and then obtained a caching hit rate that was better than some caching strategies similar to them. Considering the hidden association of user requests in the time step, Ale et al. [98] designed an online active caching policy, established a two-way deep recurrent neural network model to predict the content request of the time series, and used the fully connected neural network to learn and predict from the samples. Most of the existing research work is based on global content requests to estimate content popularity. Jiang et al. [99] proposed an online content popularity prediction algorithm based on content features from the perspective of local users, using a logistic regression model to approximate user preference models.

Some researchers use machine learning for modeling, but traditional machine learning does not dynamically adjust the training model based on new samples collected. In a constantly changing environment, more researchers choose to use reinforcement learning for modeling. However, users can generate content anytime and anywhere, which will result in a large amount of “unpopular” content, which cannot be deployed and transmitted or differentiated by comparison of popularity. The marginalization of user content and the self-organizing transmission between edge nodes will also make it difficult for the central server to obtain information requested by users [100].

### 4.2. User Model Analysis

In order to design more targeted caching policies for different users or groups, analyzing and establishing a user model that conforms to real mobile users is also significant in the delivery process. Therefore, we can predict the mobile user trajectory and model the mobile user behavior in advance, and then cache the content that the user will request in advance on the BS in the user movement path. Mobility models can usually be divided into random mobility models, time-related mobility models, and space-related mobility models [101], and can also be further divided into entity mobility models, group-based mobility models, human or sociality-based mobility models, and vehicular mobility models [102].

The information on user mobility attributes also helps to analyze and build user models, including temporal properties and spatial properties [103]. Temporal properties are features related to time, and spatial properties include information about the user geographic location and the user movement pattern. Considering the information on user mobility attributes, Conan et al. [104] modeled the contact model between users as a Poisson process to obtain the average contact time between users in a self-organizing network. Lee et al. [105] proposed a method to obtain the migration probability matrix and residence time distribution through the user movement-related records. In the literature [106], the user’s movement is visualized through the user movement trajectory, and the user actual movement trajectory is modeled as a random waypoint model. Because it is impossible to specify the user movement path between each unit, the transmission information of the mobile user is also particularly important. Lee et al. [105] used the Markov chain model to capture the spatial information of mobile users. In the mobile network, the movement of MDs is often closely related to the movement of users, and user behavior is more affected by people’s social attributes and habits. When the nodes in the wireless network model move, the average user throughput will increase significantly [107]. Therefore, establishing an accurate user movement model helps to improve the efficiency of the caching strategy. Hosny et al. [108] used a probabilistic random walk model to capture the mobility of a single user. In the literature [109], a Markov chain is used to model the user’s movement and predict the probability of user move, as well as consider the frequent switching of users between small units to reduce the load of the macro unit as much as possible. To determine the caching scheme for decentralized caching networks, Ye et al. [110] designed a mobile decentralized regular multi-task learning (DRMTL) model, which can more accurately predict the preference of the user geographic location. Kwshavarzian et al. [111] grouped small units into unrelated clusters and modeled the user’s movement between clusters as a Markov chain model to reduce the total energy consumption of content delivery. At the same time, some researchers are studying the user movement model in the unmanned aerial vehicle (UAV) scene [112].

By combining social networks with wireless communication networks, the virtual extension of social relationships in the network is realized, which is also a method of analyzing user models. Users with greater personal influence will have a greater impact on caching decisions, and the obtained content is more likely to be requested again by users in the same social network [86]. By modeling the characteristics of these users, the delivery process can greatly reduce the characteristic data that needs to be processed while ensuring a certain efficiency. Bernardini et al. [113] speculated that a small number of more influential users can dominate group activities. There are also some works to estimate the similarity between users by using the mobility and social relationships between mobile users [114]. In the paper [115], the author analyzed the user model based on the similarity of user interest and opportunity communication. Cai et al. [114] used the Indian self-service process to model the impact of the same content requested between devices, and then independently made caching decisions based on the level of user content contribution. Qian et al. [116] modeled the strength of social relationships through the strength of connection and similarity of interest between users and constructed a social relationship graph based on user mobility and social networks.

In this part, we must first solve the challenges that user mobility brings to the establishment of users’ models. At the same time, user mobility is also related to the social relationship between users. By determining the common ground and social relationship between users, it is more conducive to analyzing user models. Therefore, with the help of the characteristics of user mobility and social relationships, it is possible to analyze and establish a more appropriate users’ model faster.

### 4.3. Content Retrieval

When mobile users request content from the wireless network, they need to retrieve content items through the network, find the requested content items with the help of neighboring devices or caching nodes and determine the method of retrieving the content requested by the user and the caching location [117]. The first requesting user will first send the search request to the nearest user, and the two will establish communication. If the user does not cache the corresponding content locally, a search request will be sent to other nearby users. If the requested content is not found within the effective range of D2D communication, the search continues to be sent to SBSs or MBSs. Then, if the content is not cached in BSs, the request will be forwarded to the cloud center. If all requests fail, the content of the request will be uploaded to the service provider.

Considering the transmission bandwidth capacity limitations of SBS in densely populated areas, Poularakis et al. [118] proposed algorithms with approximation guarantees, which combine content placement and user request issues to maximize SBSs’ handling of user service requests. They also combined user requests with different needs and content placement issues to ensure that operators optimize service costs based on user priorities and minimize user delay [119]. However, the diversity of user needs determines the impact of different caching strategies and layered coding on delay and service costs to varying degrees, and the trade-off cost and delay will also be affected by network load. Pantisano et al. [120] considered the impact of interference and the transmission capacity of the backhaul link and stipulated that SBSs determine which users to serve based on the cached content and the user’s transmission data rate. They used a one-to-many matching game to describe the matching problem between SBSs and UEs, proposed an algorithm based on the delayed reception scheme, and completed the matching of SBSs and UEs with a reasonable number of iterations. However, they all acquiesce that SBS only serves one user at a time, ignoring that SBSs need to serve a cluster simultaneously.

Takeda et al. [121] improved the traditional content sharing scheme in peer-to-peer networks. Each peer and caching node keeps cached contents and historical retrieval records, and users can retrieve content more quickly based on historical retrieval records under the condition of ensuring the content discovery ratio. In the meantime, it will decide whether to cache the content item and share it in the system according to the popularity of the content item and the priority of the caching node. However, the experimental results show that the program does not significantly reduce the cost of content retrieval and transmission. Most of the work considers the optimization strategy of user-related nodes and content storage problems, but the additional traffic overhead and user collaborative retrieval have not yet been resolved, and further research is still needed.

### 4.4. Content Delivery

This part is mainly to solve some of the problems faced when sending cached content to users. In existing work, most of the impact of edge caching policies on content delivery has been ignored. Fang et al. [122] proposed an edge caching policy for intelligent content delivery. They use smart routers deployed at the edge of the network to analyze content popularity, user mobility, social networks, and historical access records, then make caching decisions, and update the status of network cached content promptly. However, the author did not evaluate the feasibility of the scheme in a heterogeneous network environment. In the dynamic VOD content distribution scenario of the subway network, Ayoub et al. [123] compared the network resources needed to access the network in different caching placement strategies. The results show that proper deployment of caching nodes between the access and the metro network segments can improve system performance and also affect the number of requests from routers to cloud servers. In order to adapt to changes in content requirements, Dealmeida et al. [124] proposed a CDN model. They used the Q-learning algorithm to weigh the network cost and caching hit ratio to determine the time to live (TTL) of the cached content and automatically extended the caching node to set the best TTL for the cached content. Experimental results show that the TTL meets cost optimization and achieves the lowest acceptable caching hit ratio. However, they only considered that the Internet content is stored on the local caching node and also ignored the transmission cost between the cloud server and the caching node.

**Figure 8:**
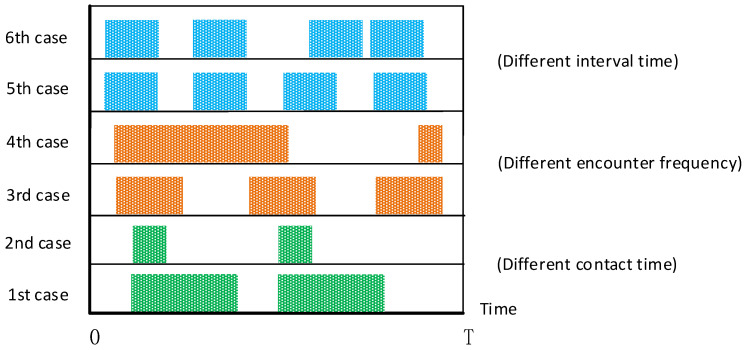
Different scenarios encountered assuming two users.

User mobility can provide an opportunity for communication between different users. In the range of D2D communication, when a mobile user passes by, in addition to obtaining the requested resource from the BS, the user can also use the local cache of nearby users to assist in downloading. Therefore, content delivery can be completed more efficiently using the user mobility. The opportunity realizes a self-organizing network of communication between nodes, without a complete communication link between the source node and the target node. Figure 8 shows a different encounter scenario assuming two users. In the time range of T, the shaded part represents the meeting time between users within the D2D communication range, and the blank interval is the separation time when D2D communication is not allowed between users. In practice, both the frequency of encounter and the time of contact need to be considered at the same time. In the first and second cases, the encounter frequency is the same and the contact time is different, and in the third and fourth cases, the encounter frequency is different and the contact time is the same. It can be clearly seen that the higher the frequency of encounters and the longer the contact time, the better the communication opportunities. In the fifth and sixth cases, the encounter frequency and contact time are the same, but the encounter interval time is different. Comparing their shortest separation time, the sixth case is better than the fifth case. In the past research work, the main indicators for estimating the strength of encounter opportunities between users are the frequency of encounters, total contact time, and contact interval time [125]. However, Bulut et al. [126] pointed out that these indicators are insufficient in measuring the intensity of user encounters.

In vehicle content delivery networks (VCDNs), exploring the provision of vehicle content delivery between vehicles and RSUs, Su et al. [84] proposed a dynamic content caching strategy based on cross-entropy. This strategy is based on the cooperation between the vehicle’s request and RSUs and selects the appropriate RSUs to place the cached content by evaluating the relative delay of moving vehicles and RSUs in retrieving the requested content. This solution effectively reduces the transmission delay and caching cost, and improves the caching hit ratio. Fang et al. [127] proposed a cooperative caching strategy for RSUs. To reduce the average download delay of VCDNs on two-way roads with a T-junction, they adopted a fast simulated anneal algorithm for content distribution to alleviate the caching capacity constraints of RSUs. However, they did not consider the impact of heterogeneous networks and regional switching of vehicles, and did not formulate a reasonable incentive mechanism to ensure collaboration.

### 4.5. Content Update

The content frequently requested by the same type of users at different times will change, and users in the same area will continue to flow in and out. The content frequently requested by users in the area will also change, and the data required by real-time applications will also be updated regularly. At the same time, the current network status cannot be accurately obtained, which will reduce the performance of the caching strategy to varying degrees. A timely caching update is crucial in edge caching. For the content update scheme, Ahlehagh et al. [128] used the least recently used (LRU) caching strategy to update, and Hassine et al. [129] used the least frequently used (LFU) caching strategy to update the content. LRU will replace the least recently used content with new content, which has been widely used in the past and used as a benchmark for the performance evaluation of caching strategies. Moreover, LFU is more complicated than LRU to calculate content popularity based on the frequency of each content request and then replace the cached content [130]. Megiddo [131] proposed an adaptive replacement caching (ARC), which can work together without considering the size of the cached content and prior knowledge. It used online and self-adjusting methods to adaptively and continuously maintain a dynamic balance between content popularity and request frequency, and constantly adapted to modify replacement standards.

In the small cell heterogeneous architecture, Bharath et al. [132] counted the cache of related and unknown popularity profiles and proposed a cached content update algorithm that captures the rate of change of popularity profiles. Jiang et al. [133] proposed a distributed deep reinforcement learning caching policy to predict users’ preferences and content popularity. They set a specified update time and combined it with real-time content update optimization in the caching strategy to improve the caching hit ratio. Huang et al. [134] studied the optimal random caching policy based on segmentation and proposed a truncated random caching policy and a low-complexity suboptimal scheme. Song et al. [135] determined the first cache order of files based on Zipf’s law based on their research work and proposed a dynamic update strategy based on a file segmented caching policy. However, MDs normally are limited by bandwidth and cannot respond to too many requests in a short time. Considering the limitations of user equipment service capacity and mobility, Jiang et al. [136] proposed a caching strategy for heterogeneous networks. They planned the caching strategy as a mixed-integer linear programming problem and then used the Lagrangian relaxation and layered a primitive dual decomposition method to solve it, which can minimize the system cost.

In this part, we mainly discuss the existing research works on the cached content update, from solving the problem of content replacement to improve the efficiency of the caching strategy. Two common content replacement strategies, LFU and LRU, have been applied in many research works, and their effectiveness has also been confirmed. In the face of the more complex network architecture and the diversity of content, we need to more accurately estimate the popularity of the content to provide strong support for cached content updates. In the cached content update, how to update cached contents is of course important, but in the meantime, we also need to consider the update frequency. Too high update frequency will increase the burden of network transmission and is not conducive to improving network efficiency. Therefore, in the next research work, it is necessary to consider how to optimize the update frequency in the meantime.

## 5. Research Challenges and Future Directions

In order to better utilize the potential of edge caching in MEC, we must consider the unique challenges in the MEC network framework and make full use of characteristic advantages to design caching strategies. In this section, we discuss some of the challenges that edge caching still faces, and point out more promising research directions.

### 5.1. Caching in IoV

With the rapid growth of the number of vehicles, the IoV has become a hotspot of research and development by virtue of its comprehensive advantages and huge potential [137]. The popularity of IoT [138] and artificial intelligence technology [139] laid the foundation for the development of intelligent transportation systems. With the impact of large-scale data generated by the emergence of smart vehicles on the network performance of the IoV and the energy consumption of vehicle users, edge caching technology has become a more promising solution to the above problems [140].

**Figure 9:**
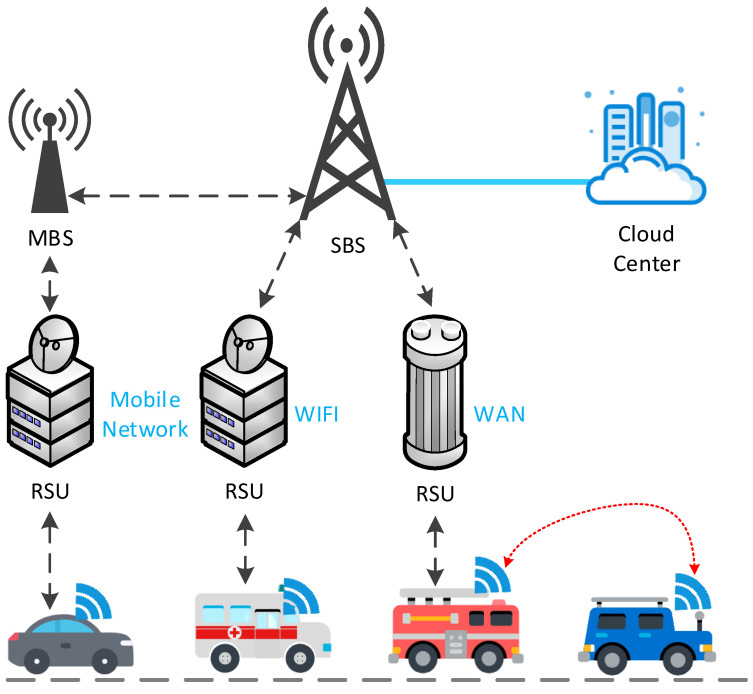
Edge caching in IoV.

Zhang et al. [141] introduced edge caching technology in the IoV, using cache-enabled smart vehicles and RSU as caching nodes, using the relationship between the two for deep reinforcement learning, and proposing an edge caching policy with social awareness. They proved that in the inter-regional and multi-vehicle social network of vehicles, the efficiency of content distribution can be maximized with low latency. In fact, different vehicles will have different social characteristics, and the communication environment between vehicles is dynamic and complex, so using edge caching in the IoV and making the network more efficient still face some difficulties. As shown in Figure 9, most of the existing caching policies cannot meet the needs of the IoV scenario. Because the social relationship and characteristics between car owners cannot be fully applied to vehicles, how to arrange services for vehicles through social attributes is also an unexplored problem. Moreover, the moving speed and driving path of the vehicle change rapidly, and it is difficult to realize the mobility-aware strategy for mobile users in the IoV. At the same time, how to motivate vehicles to follow the resource scheduling of the vehicle network and share vehicle driving information is a topic worthy of discussion.

### 5.2. Mobility Management

In wireless communication networks, when mobile users move between different cells, to ensure the continuity of network services, there are strict handover procedures for connections with different BSs [142]. Similarly, if the cached content is stored in caching nodes, it is a question of how to ensure that multiple users continuously receive the requested content during the movement and the quality of users’ experience when receiving the collaborative cached content. However, in the research work on edge caching, few go deep into this aspect. Based on the existing technology, a more realistic challenge is how to more accurately predict the action trajectory of users after requesting content, and the nearby caching nodes react in advance, so as to make the user not feel service interruption as much as possible, thereby improving the quality of users’ experience. This puts forward higher requirements for low-latency technology and motion trajectory prediction technology. It is believed that with the continuous deepening of research, these problems can be solved more comprehensively.

### 5.3. Security and Privacy

Security and privacy of data are technical difficulties that need to be considered in edge caching. The security of caching nodes’ storage content and privacy issues related to contents of users’ information. Because edge caching faces more frequently changing wireless channels and mobile traffic, many of the solutions that have been proposed for file security and privacy cannot be applied. Therefore, it is still necessary to further develop a special solution for the special situation of edge caching.

In Content-Centric Networking (CCN), cached content is often named in combination with the content itself, so the content cached in the node hides a large amount of communication information and action tracks of users. Hackers only need to obtain the caching list, and they can pretend to request the corresponding cached content. In this way, the user’s private information is obtained by others [143]. Users can also determine whether the content is in a specific caching node by detecting the response time of a specific content, and then obtain the privacy information of nearby users and monitor behaviors and trajectories of nearby users [144]. In the existing research work, by grading content’s privacy and users’ security, the obvious differences caused by different characteristics of cached content are used to classify security levels [144], and anonymous sets are used to protect private information [145], etc. They are constantly weighing caching performance and security, and they only conduct security simulation experiments under artificial data. How to not only ensure the security and privacy of cached contents but also meet the needs of users for caching performance is a major challenge facing edge caching.

### 5.4. Fading and Interference

Channel fading and interference between cooperative caching nodes are also key issues that need to be solved urgently in edge caching. The non-negligible path loss and the unavoidable mutual interference problem when the same user receives cached contents between cooperative nodes, as well as the complex interference distribution in heterogeneous networks [81], will limit the network capacity and transmission efficiency. This also leads to a high enough cache hit ratio, but the performance improvement of the cache system is still limited. When caching nodes near the user do not cache the content requested by the user, the user needs to request a service from a node farther away. However, the interference is often stronger than the signal strength, and near nodes will interfere with the service of the user by the far node. Therefore, when we study the layout and mobilization mechanism of caching nodes, we must consider interference issues.

In the work of [28,146], a user-centric inter-cell interference nulling strategy was proposed, and SBSs within the interference range of each user can also be used to coordinate data transmission to users [147]. Others have proposed to use conflict graphs to consider routing and channel allocation in wireless network nodes, thereby solving the problem of channel interference [148]. To make full use of the storage resources of caching nodes, reduce caching overhead and increase STP, we still need to conduct further research on channel fading and interference.

### 5.5. Node Storage Capacity

Due to the limited storage resources of BSs and terminal devices, the storage capacity of caching nodes also needs to be considered. Especially in the D2D caching strategy, the storage capacity of MDs is constantly changing over time, and the local caching space that users are willing to share will also be affected. This makes the research of a caching strategy more complicated. At the same time, it is also necessary to consider the matching of the storage capacity of caching nodes and the connection capacity of nodes and make full use of the available storage capacity of a caching node to maximize the edge caching hit ratio when the node connection capacity is sufficient. This requires us to be able to grasp the storage capacity information of caching nodes at all times and make corresponding decisions in time to actively mobilize resources. In the work of [149], a capacity-aware edge caching strategy was proposed, which is to allow the collaboration between the caching node and the cloud server and cache appropriate content in caching nodes according to the limited storage resources of the node and the connection capacity of the node and the user. However, the environment of the network is dynamically changing. Channel fluctuations, cached contents of different sizes, and the performance of devices held by users need to be considered. Further research is needed to develop more practical caching policies that consider the storage capacity of caching nodes.

### 5.6. Incentive Mechanism

The D2D caching strategy is to reduce remote network data transmission and transmission waiting delays by sharing local caches between adjacent users and caching nodes, as well as the caching time of popular content is controlled by the mobile network to avoid caching content during peak periods and reduce the burden on the backhaul link [150]. Therefore, its performance is mainly affected by the willingness of users and caching nodes to share. Therefore, the incentive mechanism plays a very important role in a deeper sinking caching strategy. However, due to the large amount of personal privacy information stored in MDs and limited battery life, most users are unwilling to share local storage resources and actively join D2D caching. How to motivate users to voluntarily share local content and assist the operation of the D2D caching system is an important challenge. In the next research work, it is also necessary to consider how to make full use of the caching resources of the edge device and encourage users to actively assist the edge cache in the case of asymmetric information.

## 6. Conclusions

With the explosion of live broadcasts and short video applications, and the popularization of 5G networks from Non-Stand Alone to Stand Alone, the need for lower latency and higher network throughput of wireless communication networks is imminent. Edge caching technology provides a new idea beyond traditional communication technology for how to better combine user preference content and mobile user characteristics for Internet content transmission under the existing resources. It can reduce the repeated data transmission in the backhaul link and improve the quality of the mobile user experience. Our work is mainly to conduct a comprehensive investigation of all aspects of edge caching and point out the importance of edge caching technology. We summarize related work of caching placement optimization from different stakeholder perspectives. Then, we discussed caching policies from different caching methods, and pointed out the problems that need to be solved through comparative analysis. In particular, we have discussed the delivery process, summarized as five phases, including requested content analysis, user model analysis, content retrieval, delivery, and update, indiviudally. Finally, we put forward several challenges and potential future directions, including the application in IoV, the influence of edge device characteristics, etc., and hope to bring some ideas for the follow-up researches in this area. Meanwhile, recent studies generally use some data sets of simple scenes for simulation and analysis to verify the proposed scheme. Nevertheless, to demonstrate the expected effect introduced by the edge caching, actual tests and trials under more realistic assumptions are further required.

## Data Availability

Not applicable.

## Abbreviations

The following abbreviations are used in this manuscript:

5G	5th Generation Mobile Networks
CDN	Content Delivery Network
MIMO	Multi-Input Multi-Output
BS	Base Station
MBS	Macro Base Station
SBS	Small Base Station
MCC	Mobile Cloud Computing
MEC	Mobile Edge Computing
D2D	Device-to-Device
MAC	Media Access Control
MD	Mobile Device
ISP	Internet Service Provider
SIR	Signal to Interference Ratio
PPP	Poisson Point Process
QoE	Quality of Experience
QoS	Quality of Service
DTX	Discontinuous Transmission
STP	Successful Transmission Probability
OFDM	Orthogonal Frequency Division Multiplexing
VOD	Video-on-Demand
MAB	Multi-armed Bandit
MDP	Markov Decision Process
TTL	Time to Live
VCDN	Vehicle Content Delivery Network
RSU	Roadside Unit
LRU	Least Recently Used
LFU	Least Frequently Used
ARC	Adaptive Replacement Caching
IoV	Internet of Vehicles
CCN	Content Centric Networking
